# Gene-Therapeutic Strategies Targeting Angiogenesis in Peripheral Artery Disease

**DOI:** 10.3390/medicines5020031

**Published:** 2018-03-30

**Authors:** Fumihiro Sanada, Yoshiaki Taniyama, Jun Muratsu, Rei Otsu, Hideo Shimizu, Hiromi Rakugi, Ryuichi Morishita

**Affiliations:** 1 Department of Clinical Gene Therapy, Osaka University Graduate School of Medicine, Suita, Osaka 565-0871, Japan; taniyama@cgt.med.osaka-u.ac.jp (Y.T.); muratsu@cgt.med.osaka-u.ac.jp (J.M.); otsu@cgt.med.osaka-u.ac.jp (R.O.); taropapa@mx6.nisiq.net (H.S.); 2 Department of Geriatric and General Medicine, Osaka University Graduate School of Medicine, Suita, Osaka 565-0871, Japan; rakugi@geriat.med.osaka-u.ac.jp

**Keywords:** angiogenesis, gene therapy, hepatocyte growth factor

## Abstract

The World Health Organization announced that cardiovascular disease is the number one cause of death globally, representing 31% of all global deaths. Coronary artery disease (CAD) affects approximately 5% of the US population aged 40 years and older. With an age-adjusted prevalence of approximately 12%, peripheral artery disease (PAD) affects at least 8 to 12 million Americans. Both CAD and PAD are caused by mainly atherosclerosis, the hardening and narrowing of arteries over the years by lipid deposition in the vascular bed. Despite the significant advances in interventions for revascularization and intensive medical care, patients with CAD or PAD who undergo percutaneous transluminal angioplasty have a persistent high rate of myocardial infarction, amputation, and death. Therefore, new therapeutic strategies are urgently needed for these patients. To overcome this unmet need, therapeutic angiogenesis using angiogenic growth factors has evolved in an attempt to stimulate the growth of new vasculature to compensate for tissue ischemia. After nearly 20 years of investigation, there is growing evidence of successful or unsuccessful gene therapy for ischemic heart and limb disease. This review will discuss basic and clinical data of therapeutic angiogenesis studies employing angiogenic growth factors for PAD patients and will draw conclusions on the basis of our current understanding of the biological processes of new vascularization.

## 1. Introduction

Human muscle, including that in lower extremities and the heart, has an innate capacity to remodel in response to advancing artery disease [1]. With the progression of atherosclerotic plaque in the major arteries, patients gradually begin to grow small collateral blood vessels to overcome restricted blood flow and improve organ perfusion. This process is known as angiogenesis [2]. Collateral blood flow after major artery occlusion may be sufficient in some patients to meet ischemic skeletal muscle or myocardial needs at rest. However, collateral circulation is generally not sufficient to meet oxygen consumption during exercise [3], which profoundly limits patient’s physiological activity and quality of life. These “refractory ischemia” patients are no longer responsive to anti-anginal and anti-platelet medications. They are either not candidates for stent implantation or bypass surgery, or continue to suffer from angina/muscle pain even after these mechanical revascularization procedures [4]. While drug and proteins appear unsuitable, new research and clinical studies focused on angiogenic gene therapy are now showing some progress in the treatment for peripheral artery disease (PAD).

Researchers have long been faced with the challenge of amplifying the primal angiogenic healing response and regulating it through the design and development of angiogenic therapeutics [5,6]. For about 20 years, several attempts targeting angiogenesis have been developed including cell therapy and gene therapy. Cell therapy is still at the primitive stage, requiring randomized large placebo control studies [7]. Although gene therapy targeting angiogenesis for coronary artery disease (CAD) and PAD is still at an early phase, several clinical trials using angiogenic growth factor genes for PAD have recently been conducted, including successful and unsuccessful trials.

In this narrative review, we would like to discuss (i) angiogenic growth factors used in clinical trials that regulate the multiple signals required to orchestrate micro-vessel growth and enlargement; and (ii) gene delivery systems for angiogenic gene therapy targeting peripheral ischemic tissue.

## 2. Vasculogenesis, Arteriogenesis, and Angiogenesis

Postnatal growth of blood vessels is regulated by the following mechanisms: vasculogenesis, arteriogenesis, and angiogenesis [8]. Vasculogenesis is the de novo formation of vasculature from progenitor or stem cells. This mechanism has attracted a great deal of attention through the discovery of endothelial progenitor cells [9]. However, its role in vasculogenesis after birth is still under debate [10]. In contrast, arteriogenesis is induced by physical forces, most importantly shear stress. Chronically elevated fluid shear stress was found to be the strongest trigger under experimental conditions [11]. Studies have shown that collateral remodeling can be reversible up to a certain point of this process, in which case shear stress turns to normal after successful thrombolysis or surgical thrombectomy [11]. Thus, arteriogenesis describes the remodeling of pre-existing arterio-arteriolar anastomoses to functional arteries. Unfortunately, its efficiency dramatically decreases with disease and aging. Angiogeneis is the formation of blood vessels by the migration, proliferation, and sprouting of pre-existing endothelial cells [11]. The reduction of tissue oxygen tension induces angiogenesis response in disease conditions including CAD and PAD. Under physiological condition, capillaries are stabilized by the anti-angiogenic stimuli (TGF-β, Notch1, thrombospondin, angiostatin, etc.) that balance the effect of angiogenic growth factors such as vascular endothelial cell growth factor (VEGF), fibroblast growth factor (FGF), hepatocyte growth factor (HGF), and platelet-derived growth factor (PDGF) [12]. Reduced oxygen disrupts the normal balance toward angiogenic events. Most transcriptional responses to low oxygen are mediated by hypoxia-inducible factors (HIFs), highly conserved transcription factors that control the expression of numerous angiogenic, metabolic, cell survival, and cell cycle genes [13]. HIF-mediated genes increase endothelial cell proliferation, migration, and blood vessel sprouting, thus described as “angiogenic growth factors”. These angiogenic growth factors include soluble growth factors (VEGF, FGF, and PDGF, etc.) and cytokines, such as matrix metalloproteinases (MMPs) and urokinase (uPT), bound to the extracellular matrix, enabling endothelial cell sprouting [13]. The endothelial cells directing the vascular sprouts are known as endothelial tip cells [14]. The tip cells are trailed by the endothelial stalk cells, which are highly proliferative, create adherent and tight junctions to ensure the stability of the new sprout vessels, and form the new capillaries [15,16]. Normalized blood supply decreases the hypoxic stimuli and tip cells lose their sprouting potential, stabilizing newly formed capillaries. A recent finding demonstrated that VEGF and Dll4-Notch1 signaling is involved in an intricate cross talk to balance tip and stalk cell formation and to regulate directed tip cell migration and stalk cell proliferation [17,18]. However, it is still unclear whether all of these angiogenesis events continue to operate properly under disease conditions, including PAD. With the identification of several proangiogenic molecules, it is recognized that therapeutic interference with vasculature formation offers a tool for clinical applications in PAD.

## 3. Choice of Angiogenic Growth Factor

One key element of successful gene therapy is identifying the growth factors that are able to stimulate the complex angiogenic biological process and that can resolve the complicated pathology of critical limb ischemia (CLI), the most severe manifestation of PAD. The delivery of VEGF and FGF has been widely debated and studied. Also, the angiogenic property of HGF has been revealed. Table 1 reports the human clinical trials of angiogenic growth factors for patients with PAD.

### 3.1. VEGF

The VEGF family consists of VEGF-A to -E. VEGF-A regulates angiogenesis and vascular permeability through the activation of two receptors, VEGFR-1 and VEGFR-2. In contrast, VEGF-C and -D mainly regulate lymphangiogenesis [30]. Among VEGF-A alternative splicing isoforms, VEGF_165_ is the most abundant and best studied. In an animal study, the delivery of VEGF_165_ by plasmid or adenovirus vector significantly improved tissue perfusion with neovascularization in a hind limb ischemia model [6,31]. It has been described that VEGF-A stimulates endothelial cell proliferation and migration, as well as endothelial progenitor cell recruitment to injured site, with subsequent angiogenesis and vasculogenesis [32,33]. A clinical trial using VEGF gene transfer demonstrated inconsistent results. Isner J.M. et al. demonstrated the intramuscular injection of naked-plasmid VEGF_165_ gene transfer promotes collateral vessel formation in CLI patients [19]. Mäkinen et al. showed that the intra-arterial administration of Ad-VEGF_165_ significantly increased vascular density as compared to the placebo control group [20]. Contrary to VEGF_165_, intramuscular injection of the Ad-VEGF_121_ isoform, which is a more mitogenic isoform than VEGF_165_ or VEGF_189_, failed to show an improvement in the ankle-brachial index (ABI), intermittent claudication, and quality of life in a phase II clinical trial [21]. Moreover, intramuscular injection of VEGF_165_ plasmid (Groningen trial) showed no improvement in amputations in a phase II trial [22].

Unfortunately, to date, no phase III clinical trial has shown evidence of benefit regarding VEGF gene therapy in PAD patients. VEGF gene therapy is strongly linked to dose-dependent microvascular permeability and peripheral edema. Approximately 60% of patients developed moderate or severe edema in clinical trials. This adverse side effect is caused by the Rac 1-mediated generation of reactive oxygen species (ROS), which, in turn, regulates the adherence junction integrity [34]. Ehrbar et al. developed new delivery systems (α2PI1-8-VEGF_121_) that can control the level of VEGF in an animal model with generated non-leaky vessels and ameliorated vessel formation more potently than did native VEGF_121_ gene therapy [35]. This data suggests that long-term, slow-release, and low-dose VEGF therapy might be more effective and feasible in a clinical setting. Additionally, recent research has suggested the use of a specific regulatory gene, FGF-4, that is now known to activate VEGFs and the cascade of events required to stimulate angiogenesis, and is considered a more fruitful approach that VEGF alone [36,37]. Using a regulatory gene is likely more practical than trying to determine which individual growth factor or growth factor combination is best suited for the angiogenic gene therapy.

### 3.2. FGF

In humans, 22 members of FGF family are identified, all of which bind to four spliced isoforms of the FGF receptors, FGFR1-4. FGF signals through four receptor tyrosine kinases act in a variety of developmental processes, including angiogenesis. Of the four FGF receptor tyrosine kinases, FGFR1 has the highest level of expression in the endothelium [38]. Oladipupo, S.S. et al. documented that the endothelial-specific deletion of FGFR1 alone or together with FGFR2 has no effect on vascular development; however, it impairs the neovasculogenesis response to injury [39]. Among FGF family members, FGF-1 (acidic), FGF-2 (basic), and FGF-4 are highly angiogenic, and can stimulate blood-vessel formation to facilitate angiogenesis with the sprouting of capillaries from vessels. Based on these findings, a non-viral FGF vector (NV1FGF, a naked DNA plasmid vector) carrying human FGF-1 has been developed for gene therapy. Experimental models and early human studies successfully demonstrated its potential to generate a functional vascular network in the ischemic region [40]. Comerota, A.J. et al. organized a phase I clinical trial enrolling 51 patients with CLI, in which a significant improvement in ulcer size, claudication, ABI, and transcutaneous tissue oxygen in the ischemic limb was demonstrated [23]. The subsequent TALISMAN phase II clinical trial was conducted in 125 patients with PAD who were not candidates for revascularization. Patients underwent four cycles of eight intramuscular injections of NV1FGF or placebo. After 25 weeks, no significant difference in the primary endpoint of ulcer healing or the secondary endpoint of ABI was observed. However, there was a significant reduction in all and major amputations at 12 months [24]. With great hope, a phase III randomized clinical trial (TAMARIS) was carried out enrolling 525 patients with CLI who were not eligible for revascularization [25]. However, unexpectedly, no benefit of NV1FGF was detected in either the primary efficacy endpoint (major amputation or death) or secondary endpoints (skin lesion status, pain index, minor amputation, quality of life, and ABI). After these disappointing results, there have been no further human trials with NV1FGF gene transfer. Associations of FGF gene therapy with potential hypertension and membranous nephropathy have been reported. These adverse effects have to be carefully considered when determining the dose and duration of administration of FGF.

### 3.3. HGF

HGF, also known as scatter factor, was originally discovered as a mitogen factor for hepatocytes [41]. Later, its angiogenic potential through the tyrosine phosphorylation of its receptor, c-Met, was discovered. Although hepatocyte growth factor is secreted mainly by mesenchymal origin cells, the targets of this multifunctional growth factor are cells of both mesenchymal and epithelial origin. Indeed, the expression of receptor c-Met has been identified on fibroblasts [42], smooth muscle cells [43], as well as EC [44] and EPC [45]. Clinical trials using the HGF gene for the treatment of PAD patients are particularly noted. To date, three randomized placebo-controlled clinical trials using naked human HGF plasmid DNA proved its beneficial effect in the healing of ulcers, decrease in rest pain, and increase in trans-cutaneous oxygen tension [26,28,29]. The long-term efficacy of HGF gene therapy with an increase in ABI, and a reduction of rest pain and ulcer size 2 years after gene therapy has also been demonstrated [27]. It is also worth mentioning that, unlike VEGF, HGF gene therapy is not associated with edema nor any adverse side effects. Very recently, a biopharmaceutical company submitted an application for marketing approval to the Pharmaceuticals and Medical Devices Agency (PMDA) of Japan for HGF plasmid. Among HGF’s beneficial functions, its anti-inflammatory and anti-fibrotic effect differentiates HGF from other angiogenic growth factors. An HGF/cMet system can attenuate Angiotensin II-induced ROS production through ligand-dependent epithelial growth factor receptor (EGFR) degradation. This mechanism is also operated following the administration of endothelin-1 (ET-1), lipopolysaccharide (LPS), and transforming growth factor beta (TGF-β), which trans-activate EGFR, suggesting that the ligand-dependent EGFR downregulation of HGF might be the major anti-inflammatory and anti-oxidant mechanism of HGF [43,46]. This effect was not observed with VEGF. Also, bFGF alone, but not HGF, significantly activated a fundamental transcription factor for inflammation, NFκB, and the gene expression of its downstream inflammation-associated cytokines (IL-8 and MCP-1) in vascular smooth muscle cells, accompanied by an increase in vascular permeability in a rat paper disc model [47]. In contrast, the expression of VEGF increases after vascular injury, recruiting monocyte-lineage cells and thus enhancing neointimal formation [48]. Importantly, HGF was shown to have a synergistic action with VEGF on EC proliferation and chemotactic response and neovascularization [49,50]. HGF significantly increased VEGF expression with the reduction of VEGF-induced NFκB activation and leaky vessels or edema. Thus, HGF stimulates angiogenesis accompanied by the inhibition of the complication of inflammation, edema, and cellular senescence, which are main pathologies of CLI. The anti-fibrotic action of HGF might also contribute to resolving the complications of CLI, leading to better tissue oxygenation [51,52,53,54].

## 4. Choice of Gene Delivery System 

Aside from an understanding of the character of individual angiogenic growth factors, another separate question remains. What is the best suited DNA delivery system for angiogenic gene therapy?

### 4.1. Delivery Route

It is impossible to quantify and measure the distribution of angiogenic gene delivery in human studies. Therefore, finding the best route to deliver the plasmids or viral vectors is a significant limitation. In animal experiments, systemic administration of naked plasmid from the tail vein results in an effective gene transfer to the liver [55]. Systemic delivery from the vein might lead to a non-specific gene transfer to the liver before reaching the target organ. Naked plasmid or adenovirus vector can be delivered to skeletal muscle via an intra-arterial route. After intra-arterial infusion, vectors seem to be transferred to muscle tissue via the high density of capillaries in the vicinity of myofibers [55]. However, intra-arterial injection might also result in non-target effects in a broad area of ischemic region. The local delivery of gene therapy via intramuscular injection can also be considered for gene transfer to skeletal muscle. Intramuscular injection can deliver genes more specifically in the target regions, but may result in limited or insufficient gene delivery. Therefore, vectors encoding an angiogenic factor could be directly or indirectly delivered to ischemic muscle tissues via intra-arterial or intramuscular routes for therapeutic angiogenesis. However, currently the optimal site and route of delivery is unknown due to the inability to measure the distribution of injected vectors.

### 4.2. Plasmids or Viral Vectors

In a preclinical non-ischemic animal model, it is implicated that angiogenesis might need to be induced for weeks or months before the newly formed vasculatures mature [56,57]. Thus, the failure of initial protein therapy for CAD is attributed to the short half-life of angiogenic protein that limits target cell exposure to angiogenic stimuli. Vector systems that meet this requirement include plasmids and viral vectors. Table 2 reports the gene delivery procedures that are currently available for gene therapy in the PAD area. Plasmids are safe and easy to manufacture. Moreover, skeletal muscle and cardiomyocytes are capable of spontaneously taking up naked plasmid DNA, although the mechanism by which this occurs is still unclear. However, plasmids have a low level and short duration of muscle transduction by direct intramuscular injections due to the very low efficiency at which DNA internalization occurs. In fact, the duration of transgene expression is usually less that 10–14 days following gene delivery by plasmid. Contrary to common belief, long-term foreign gene expression from naked plasmid DNA is possible even without chromosome integration if the target cell is post-mitotic or slowly mitotic, as in skeletal muscle, and if an immune reaction against the foreign protein is not generated [58]. The utilization of ultrasound, which creates physical pores in the cell plasma membrane, is a novel way to improve the delivery of plasmid DNA into muscle or endothelial cells [59,60]. Naked DNA can penetrate into the cell plasma membrane through the pores. This method is still at the stage of development and further optimization is required for clinical application. The achievement of appropriate levels of gene expression and an optimized period of time of target gene expression following plasmid gene transfer in humans have to be answered in order to achieve successful angiogenic gene therapy. In this regard, adenoviral vectors have been shown to achieve high transfection efficiency in skeletal and heart muscle cells with transgene expression lasting for 2 to 6 weeks. The relatively short duration of growth factor gene expression by the adenovirus serotype 5 (Ad5) vector has proved sufficient for the building of new functional biological structures such as coronary collateral vessels in an animal experiment [61]. However, significant concerns regarding the safety of adenovirus gene delivery has been raised after an 18-year-old man with an inherited enzyme deficiency died 4 days after a genetically altered adenovirus vector injection into his liver intravascularly [62]. In this single case, adenovirus vector triggered significant immune response, systemic inflammatory response syndrome, and disseminated intravascular coagulation. Moreover, intravenously injected Ad5 causes a rapid hemodynamic response presenting with hypotension, cardiac output suppression, hemo-concentration, tissue edema, and vasocongestion [63] due to the upregulation of platelet-activating factor in macrophages, a known shock-inducer lipid signaling molecule [64]. Similarly, it is evidence that much more unmethylated CpG motifs in plasmids than in eukaryotic cell DNA interact with tall-like receptor, resulting in the activation of immune response [65]. Viral vectors based on the adeno-associated virus (AAV) appear particularly suitable to address the more efficacious and tissue-specific gene delivery, since they display a specific tropism for skeletal muscle cells or cardiomyocytes, and drive the expression of the therapeutic genes in these cells for indefinite periods of time in post-mitotic tissues [66]. The lack of inflammation or immune response at the site of injection of AAV enables transducing cells at high titer viruses and with multiple infections [67]. Given these favorable characteristics for gene delivery, it might be reasonable to assume that AAV will become a more frequently used vector in clinical cardiovascular gene delivery in future. Despite the remarkable progresses made over the last several years in gene delivery for the production of therapeutic factors in the cardiovascular area, a more careful investigation of the limitations of gene delivery vectors should be conducted and the appropriate duration and dosage of plasmid and viral vectors have to be addressed to gain the potential benefits of gene therapy. Large animal models are necessary to determine the optimal therapeutic agent, dose, and clinically relevant delivery strategy.

## 5. Future Perspectives

As summarized above, we now recognize that the dramatic efficacy of single-angiogenic growth factor gene therapy that was shown in many preclinical animal experiments was not fully translated into clinical practice, especially in late-stage clinical trials. Of course, a comprehensive understanding of the basic biology of neovascularization in pathological conditions would provide critical information for future successful therapeutic angiogenesis. Still, there are several obstacles to be considered for successful cardiovascular gene therapy. For instance, most of the preclinical studies use hind limb ischemia model in healthy rodents, who have no cardiovascular risk factors. Patients with PAD normally have hypertension, hyperlipidemia, and/or diabetes, which increase tissue inflammation, fibrosis, and cell senescence. Thus, gene therapy needs to dissolve complexes associated with PAD. Moreover, mimicking the aspects of human PAD in one animal model is not feasible, because PAD in humans progresses chronically, whereas the most of the animal models employed are acute ischemia models. The difference is critical with respect to the amount of collateral circulation development. Chronic ischemia in humans generally demonstrates a network of collateral vessels that are somewhat protective. With acute occlusions in animal models, there are no alternative routes of perfusion available, thus urgent and timely angiogenesis can preserve limb viability. Understanding the difference between acute and chronic ischemia and employing appropriate animal models for chronic hind limb ischemia might help to develop a new therapeutic approach for the treatment of PAD. Additionally, patient selection might have affected the results of clinical trials. Normally, patients with no options for treatments were included in late-stage clinical trials. Patients with less severe disease conditions, such as patients who would be additionally treated with revascularization procedures, may benefit more from angiogenic gene therapy. Endpoint selection is another critical point, because some endpoint measurements might be affected by the placebo effect.

In spite of significant advances in medicine, interventional therapy, and surgical therapy, peripheral arterial disease patients are increasing with the aging world population [68,69]. To address this unmet need, scientists have accumulated data on therapeutic angiogenesis by gene therapy for more than 20 years from clinical and preclinical studies. Although it is challenging to translate basic research to the clinical situations, the lessons from preclinical and clinical studies might offer better therapeutic development.

## Figures and Tables

**Table 1:** Human clinical trials of angiogenic growth factors for patients with peripheral artery disease (PAD).

Trials [Reference]	Vector and Promoter	Delivery Route	Phase	Enrollment	Outcomes
Baumgartner et al. [19]	phVEGF165/MIEhCMV	Intramuscular	I	9	Tolerated
Makinen et al. [20]	phVEGF165/MIEhCMV	Intra-arterial	II	54	Tolerated, increased vascularity
	AdVEGF165/MIEhCMV				
RAVE [21]	AdVEGF121/MIEhCMV	Intramuscular	II	95	No improvement of exercise performance or QOL
Groningen [22]	phVEGF165/not reported	Intramuscular	II	54	No reduction in amputation rate
Comerota et al. [23]	phFGF-1/MIEhCMV	Intramuscular	I	107	Tolerated
TALISMAN [24]	phFGF-1/MIEhCMV	Intramuscular	II	125	Reduction in amputation rate
TAMARIS [25]	phFGF-1/MIEhCMV	Intramuscular	III	525	No improvement of QOL or ABI, no reduction in amputation rate or death
Morishita et al. [26]	phHGF/MIEhCMV	Intramuscular	I/IIa	22	Tolerated
Makino et al. [27]	phHGF/MIEhCMV	Intramuscular	I/IIa	22	Improvement of ABI, reduction in rest pain and ulcer size up to 2 years
HGF-STAT [28]	phHGF/MIEhCMV	Intramuscular	II	104	Improvement in TcPO2
TREAT-HGF [29]	phHGF/MIEhCMV	Intramuscular	III	40	Improvement in rest pain and ABI, reduction in ulcer size

ABI: ankle-brachial index, TcPO2: transcutaneous oxygen tension, MIEhCMV, major immediate-early enhancer/promoter from human cytomegalovirus.

**Table 2:** Gene delivery system.

Strategy	Methods	Advantage	Disadvantage
Naked DNA plasmid	Direct injection	Easy to produce and use	Low efficacy, transient expression
		Low cost	
	Plus ultrasound	Improves delivery of plasmid DNA	Not well optimized
Viral vector			
Adenoviral vectors	Direct injection/intra	High multiplicity of infection	Activation of inflammatory and immune response
	Vascular injection	High levels of expression	Transient expression
		High cloning capacity	
		Broad cell tropism (infection of both quiescent and proliferating cell)	
Retroviruses vectors	Direct injection	Long-term gene expression	Insertional mutagenesis
(Lentivirus and retrovirus)		Low immunogenicity	Limited cloning capacity
		Integrative in both quiescent and proliferative cells	
Adeno-associated virus (AAV) vectors	Direct injection/intra	Low immunogenicity	Limited cloning capacity
		Site-specific integration	Difficult to produce pure viral stocks
	Vascular injection	Specific tropism for skeletal and cardiac muscle	
		Easy propagation in high titers	
		Long-term gene expression	
